# Phenolic constituents and antioxidant properties of five wild species of *Physalis* (Solanaceae)

**DOI:** 10.1186/s40529-015-0101-y

**Published:** 2015-09-18

**Authors:** José Roberto Medina-Medrano, Norma Almaraz-Abarca, M. Socorro González-Elizondo, José Natividad Uribe-Soto, Laura Silvia González-Valdez, Yolanda Herrera-Arrieta

**Affiliations:** Centro Interdisciplinario de Investigación para el Desarrollo Integral Regional, Unidad Durango, Instituto Politécnico Nacional, Sigma 119, Fraccionamiento 20 de Noviembre II, 34220 Durango, Dgo. Mexico

**Keywords:** *Physalis*, Antioxidant capacity, Phenolic profiles, Phenolic content

## Abstract

**Background:**

Fruits of wild species of the genus *Physalis* are consumed as food and calyces and leaves are used in traditional medicine. The phenolic composition of the species of this genus have been scarcely studied. To contribute to a better knowledge for the use of all the potential of these wild species of plants, leaves, fruits, and calyces of five wild species of the genus were analyzed for their phenolic composition and antioxidant properties.

**Results:**

Important tissue- and species-dependent variations were found. Calyces of *Physalis subulata* showed the highest contents of phenolics (176.58 mg of gallic acid equivalents/g dry tissue), flavonoids (39.63 mg/g dry tissue), and phenolic acids (50.57 mg of quercitrin equivalents/g dry tissue), and its leaves displayed the highest total antioxidant capacity (3.59 mg of ascorbic acid equivalents/mL) and one of the highest reduction powers (0.54 µg of ascorbic acid equivalents/mL). A high performance liquid chromatography with photodiode array detection analysis revealed a total of 28 phenolic compounds in foliar tissues (mainly kaempferol-3-*O*-glycosides), 16 in fruits (mainly phenolic acids), and 16 in calyces (mainly kaempferol-3-*O*-glycosides); the profiles of these compounds in the three types of tissue were species-specific.

**Conclusions:**

The studied species of *Physalis* are important sources of phenolics with relevant antioxidant activity. The current results indicate that phenolic profiles are valuable specific chemical markers and can be relevant as food tracing and authenticity indicators for plant-based preparations involving species of *Physalis.*

## Background

Plants are a promising source of beneficial compounds for human health and represent a starting point for the discovery and development of drugs for the treatment of human diseases (Kouloura et al. 2014). There is an impressive plant diversity worldwide, in which the chemical variation is represented by a great deal of bioactive compounds. The interest in wild plants as a source of natural antioxidants is valid as a healthier and cheaper alternative to the synthetic ones, which have been perceived as toxic and carcinogenic (Krishnaiah et al. 2011). The family Solanaceae is constituted by around 2300 species (D’Arcy 1991), which are significant sources of phytochemicals (Eick 2008). This family includes important cultivated species, such as chili (*Capsicum annuum* L.), tomato (*Lycopersicon esculentum* Mill.), and potato (*Solanum tuberosum* L.). The family also includes numerous wild species, like most belonging to the genus *Physalis*, which is the fifth largest genus of the family, including 70–90 species (Whitson and Manos 2005). Mexico, with about 50 species of *Physalis* growing in its territory, is considered the center of origin, diversity (D’Arcy 1991), and domestication of this genus (Santiaguillo et al. 1994). The fruits of many species of *Physalis* have been important elements in the culinary traditions of the people of Mesoamerica, consumed fresh or cooked, and calyces and leaves have been used in folk medicine since pre-Columbian times. Calyces are also consumed as seasoning and leaves as food (Hernández and Yáñez 2009). However, at present, only a few species are cultivated, *P. ixocarpa*, *P. peruviana*, and *P. alkekengi* are among them.

Phenolic compounds are synthesized and accumulated in practically all plant tissues. Many of these compounds, mainly flavonoids and phenolic acids, have biological properties with medical implications (Zhang and Cui 2005). The antioxidant capacity is one of the most important of these properties, because oxidation damages nucleic acids, proteins, lipids, and other macromolecules, producing cardiovascular disorders, neurodegenerative diseases, and cancer (Ames et al. 1993). Flavonoids and phenolic acids have relevant antioxidant properties (Barriada-Bernal et al. 2014). The antioxidant capacity of plant phenolic extracts depends on their concentration (Dobre et al. 2010), but also on their accumulation profiles inside plant tissues (Barriada-Bernal et al. 2014). The concentration is affected by environmental conditions, age, and phenological stage (Almaraz-Abarca et al. 2013), while the qualitative phenolic profiles are more stable and vary among different groups of plants with a species-specific tendency (Emerenciano et al. 2001).

Few species of *Physalis* have been analyzed for their phenolic composition and antioxidant properties, among them are *P. ixocarpa* (González-Mendoza et al. 2011), *P. peruviana* (Rockenbach et al. 2008; Wu et al. 2009), *P. angulata* (Ismail and Alam 2001), and *P. alkekengi* var. *franchetii* (Diaz et al. 2012). The aim of the present study was to evaluate the abundance and diversity of phenolics and the antioxidant properties of leaves, edible fruits, and calyces of five wild species of *Physalis* [*P. angulata* L., *P. patula* Mill*., P. hederifolia* A. Gray var. *hederifolia*, *P. solanacea* (Schltdl.) Axelius, and *P. subulata* Rydb.] from Durango, Mexico to determine their potential as sources of natural antioxidants. The significance of the phenolic profiles as specific chemomarkers and their potential as tool for food tracing and authenticity was also evaluated.

## Methods

### Plant material

Leaves, fruits, and calyces of mature individuals (blooming and bearing fruits) of six natural populations of *Physalis* were collected in different locations of Durango, Mexico (Table 1). Tissues of four individuals per population were randomly sampled, combined, and three pools of samples were formed and separately analyzed. Voucher specimens were deposited at the Herbarium CIIDIR. Samples were individually dried in a ventilated oven at 40 °C, and then ground in a domestic blender. The dry, ground tissues were kept in paper bags at room temperature, in a desiccator with silica, in darkness until analysis.

**Table 1:** Collection data for the Mexican studied species of *Physalis*

Sample	Curatorial number	Species	Location	Latitude N	Longitude W	Altitude (m)	Date
401	42854	*P. angulata*	Durango, Durango	24°08′15.4″	104°31′58.5″	1864	8/08/2012
407	42861	*P. hederifolia* var. *hederifolia*	Durango, Durango	23°43′24.9″	104°24′8.9″	1975	21/08/2012
412	42866	*P. solanacea*	Durango, Durango	24°14′14.6″	104°27′34″	1872	4/09/2012
413	42867	*P. patula*	Durango, Durango	24°14′14.6″	104°27′34″	1872	4/09/2012
426	42882	*P. solanacea*	Nombre de Dios, Durango	23°58′19.3″	104°19′28.2″	1841	2/10/2012
427	42885	*P. subulata*	Nombre de Dios, Durango	23°47′50.6″	103°51′9.44″	2234	5/10/2012

### Preparation of extracts

Phenolic compounds were extracted from dry ground tissues (1 g) by maceration in 20 mL of 80 % methanol (v/v), for 24 h, by shaking at 100 rpm, in darkness, at room temperature. The extracts were centrifuged (4800*g*) for 5 min at room temperature and the supernatants formed the total extracts. Different aliquots were taken to be used in the determination of phenolic content, antioxidant potential, and HPLC–DAD analysis.

### Determination of total phenolics

The concentrations of total phenolics of each sample were determined using the Folin-Ciocalteu reagent, according to Nurmi et al. (1996). The phenolic contents were calculated by using a calibration curve of gallic acid (A_760_ = 0.003[gallic acid] − 0.0241, r = 0.9985) constructed with five concentrations of this compound (0–92 μg/mL). Phenolic contents were expressed as milligrams of gallic acid equivalents per gram of dry tissue (mg GAE/g dt).

### HPLC–DAD analysis

The HPLC–DAD analysis was carried out according to Campos and Markham (2007). Aliquots (20 µL) were analyzed in a Perkin Elmer Series 200 HPLC system and a Perkin Elmer Brownlee Analytical C18 column (4.6 × 250 mm, 5 µm), using an acidified acetonitrile–water gradient. Water adjusted to pH 2.5 with orthophosphoric acid was solvent A, and acetonitrile was solvent B, mixed following the gradient: starting with 100 % A, decreasing to 91 % over the next 12 min, to 87 % over the next 8 min, to 67 % over the next 12 min, to 57 % over the next 10 min, and held at this level until the end of the 60 min analysis. Chromatograms were plotted at 260 and 340 nm. Spectral data for all peaks were accumulated in the range of 200–400 nm by using a diode array detection (Perkin Elmer Series 200). Structural information of compounds was obtained by direct comparisons of retention time (RT) and UV spectra of resolved compounds with those of standards caffeic acid (RT: 53.13, λ_max_: 239sh, 295sh, 318), *p*-coumaric acid (RT: 37.2, λ_max_: 293sh, 308), quercetin (RT: 47.05, λ_max_: 255, 268sh, 299sh, 370), quercitrin (quercetin-3-*O*-rhamnoside, RT: 38.54, λ_max_: 255, 264sh, 295sh, 348), morin (RT: 45.4, λ_max_: 254, 264sh, 298sh, 354), hesperidin (RT: 39.34, λ_max_: 284, 335sh), and naringenin (RT: 52.25, λ_max_: 289, 335sh). Structural information was also obtained from the compilations of Mabry et al. (1970) and Campos and Markham (2007). The phenolic profile of each extract was formed by all compounds resolved in their respective chromatograms. Quantitative determinations were made by an external standard method, with the commercial references quercitrin (for flavonols), naringenin (for dihydroflavonoids), and *p*-coumaric acid (for phenolic acids), by area measurements, using standard curves (area = 3 × 10^6^ + 5 × 10^7^ [quercitrin], correlation coefficient r = 0.992; area = 2 × 10^6^ + 5 × 10^7^ [naringenin], correlation coefficient r = 0.996; area = 3 × 10^6^ + 4 × 10^7^ [*p*-coumaric acid], correlation coefficient r = 0.999). The contents were expressed as micrograms of quercitrin equivalents, naringenin equivalents, or *p*-coumaric acid equivalents/g dry tissue (QiE/g dt, NE/g dt, and CE/g dt, respectively). The sum of individual flavonoid concentrations in one sample represented the total flavonoid concentration, expressed as milligram per gram of dry tissue (mg/g dt), and the sum of individual phenolic acids corresponded to the total phenolic acid concentration, expressed as milligram per gram of dry tissue (mg/g dt).

### Free radical scavenging activity

Determination of the free radical scavenging activity was carried out following the DPPH* method described by Barriada-Bernal et al. (2014). Decreases in absorbance (at 523 nm) of an initial DPPH* solution (62 µg/mL methanol) against increasing flavonoid concentrations of samples were plotted to determine, by linear regression, the efficient concentration at 50 %, defined as the amount of antioxidant needed to decrease by 50 % the initial DPPH* concentration (EC_50_). The following curve made with DPPH* between 2 and 60 µg/mL was used to estimate the DPPH* concentration (µg/mL) in the reaction medium: A_523nm_ = 0.0019 + 0.0309 [DPPH*], correlation coefficient r = 0.9996. Antiradical activities were expressed in terms of EC_50_ in microgram per milliliter (µg/mL). Quercetin and epicatechin were analyzed in the same manner as references.

### Total antioxidant capacity (TAC)

Total antioxidant capacity was evaluated according to Prieto et al. (1999). In this method, the reduction of Mo (VI) to Mo (V) by an antioxidant, forming a green phosphate/Mo (V) complex at an acidic pH is measured. TAC values were expressed as milligrams of ascorbic acid equivalents/mL (AAE/mL), which were calculated using the following curve: A_695_ = −0.2365 + 4.2133 [ascorbic acid], correlation coefficient r = 0.9987, constructed with six concentrations of ascorbic acid between 0.1 and 1 mg/mL. Quercetin (0.1 mg/mL) and epicatechin (0.1 mg/mL) were analyzed in the same manner as references.

### Iron reducing power

The iron reducing power method is based on the measurement of the formation of Fe^2+^ from Fe^3+^ in the presence of antioxidants; Fe^2+^ was estimated according to Siddhuraju and Becker (2003). Results were expressed in terms of IC_50_ in micrograms of ascorbic acid equivalents per milliliter (µg AAE/mL), calculated from the following curve of ascorbic acid: A_700_ = 25.372 [ascorbic acid] − 0.0242, correlation coefficient r = 0.9956, constructed with five concentrations of ascorbic acid between 0.03 and 0.1 mg/mL. The highest absorbance values indicated the greatest reducing capacity. Quercetin and epicatechin were analyzed in the same manner as references.

### Data analysis

All the assays were carried out for three independent pools of each sample. Data were subjected to an analysis of variance (p ≤ 0.05), and means were separated by Tukey test. Correlations between different parameters were carried out with Pearson test, by using the SPSS statistics 17.0 software. A principal component analysis (PCA), considering all the quantitative parameters, was carried out by using Past 3.0; the contribution of each parameter for the differentiation of samples was evaluated. For the different tissues (leaves, fruits, and calyces), the phenolic profiles were made up of all compounds present in the respective HPLC–DAD chromatogram. Each compound was treated as a single chemical character. A binary matrix coded by 1 (presence) or 0 (absence) formed by all individual samples vs. all resolved compounds for each type of tissue was subjected to a cluster analysis (paired group algorithm and Jaccard similarity measure) by using Past 3.0.

## Results

### Phenolic, flavonoid and phenolic acid contents

The phenolic contents are displayed in Table 2. Significant tissue and species-dependent variations were found. Among the foliar tissues, the leaves of *P. patula* accumulated the highest level of phenolics (129 mg/g dt) and among the fruits, those of *P. hederifolia* var. *hederifolia* were the richest ones in total phenolics (86.51 mg/g dt). The calyx phenolic content of *P. subulata* was the highest found in the current study (176.58 mg/g dt).

**Table 2:** Phenolics, flavonoids, phenolic acid contents and antioxidant evaluations of five wild species of *Physalis*

Species and reference compounds	Phenolic content (mg AGE/g dt)	Flavonoid content (mg QE/g dt)	Phenolic acid content (mg CE/g dt)	DPPH scavenging capacity (EC_50_) (µg/mL)	Total antioxidant capacity (TAC) (mg AAE/mL)	Iron reducing power (IC_50_) (µg AAE/mL)
Leaves						
*P*. *patula* (413)	129.06 ± 0.66 a	13.363 ± 0.01 d	4.053 ± 0.049 cd	22.96 ± 0.62 d	2.45 ± 0.08 b	0.43 ± 0.04 b
*P.* *subulata* (427)	118.01 ± 3.34 b	20.152 ± 0.033 c	5.566 ± 0.040 b	8.83 ± 0.28 f	3.59 ± 0.26 a	0.54 ± 0.01 a
*P.* *solanacea* (426)	89.83 ± 3.3 c	9.365 ± 0.050 e	4.671 ± 0.046 d	8.18 ± 0.21 f	1.80 ± 0.04 c	0.49 ± 0.04 ab
*P.* *solanacea* (412)	86.66 ± 6.32 c	6.543 ± 0.014 f	2.388 ± 0.034 e	28.21 ± 0.96 c	2.10 ± 0.10 bc	0.44 ± 0.001 b
*P. hederifolia* var. *hederifolia* (407)	58.75 ± 5.18 d	21.265 ± 0.006 bc	1.242 ± 0.075 f	59.17 ± 1.42 a	2.35 ± 0.06 b	0.11 ± 0.001 d
*P*. *angulata* (401)	40.51 ± 0.71 e	23.036 ± 0.100 a	6.706 ± 0.007 a	38.74 ± 1.04 b	2.44 ± 0.18 b	0.20 ± 0.004 c
Quercetin				5.29 ± 0.39 g	0.08 ± 0.002 d	0.09 ± 0.003 d
Epicatechin				14.33 ± 0.29 e	0.11 ± 0.005 d	0.07 ± 0.004 d
Fruits						
*P. hederifolia var. hederifolia* (407)	86.51 ± 4.03 a	4.80 ± 0.031 b	Not found	0.87 ± 0.03 f	0.59 ± 0.006 e	0.06 ± 0.002 e
*P. patula* (413)	53.14 ± 3.95 b	1.392 ± 0.007 d	7.417 ± 0.067 a	9.14 ± 0.17 b	1.07 ± 0.04 a	0.10 ± 0.001 b
*P. solanacea* (426)	46.29 ± 1.93 bc	2.499 ± 0.023 c	1.253 ± 0.020 d	3.11 ± 0.05 d	0.82 ± 0.005 c	0.06 ± 0.002 e
*P. solanacea* (412)	40.39 ± 5.32 cd	4.642 ± 0.020 b	2.773 ± 0.032 c	2.51 ± 0.18 e	0.72 ± 0.010 d	0.03 ± 0.001 f
*P. angulata* (401)	36.92 ± 2.57 cd	Not found	7.849 ± 0.050 a	3.05 ± 0.19 c	0.92 ± 0.006 b	0.02 ± 0.00 g
*P. subulata* (427)	32.29 ± 0.93 d	5.462 ± 0.033 a	4.258 ± 0.03 b	2.15 ± 0.18 e	0.58 ± 0.15 e	0.15 ± 0.001 a
Quercetin				5.29 ± 0.39 c	0.08 ± 0.002 f	0.09 ± 0.003 c
Epicatechin				14.33 ± 0.29 a	0.11 ± 0.005 f	0.07 ± 0.004 d
Calyces						
*P. subulata* (427)	176.58 ± 12.55 a	39.633 ± 0.079 a	50.570 ± 0.125 a	60.29 ± 0.30 a	2.07 ± 0.014 a	0.34 ± 0.02 b
*P. solanacea* (426)	170.78 ± 7.06 a	5.269 ± 0.059 d	5.805 ± 0.042 b	12.20 ± 0.17 d	1.56 ± 0.040 b	0.55 ± 0.03 a
*P. solanacea* (412)	76.68 ± 4.99 b	3.678 ± 0.049 e	4.054 ± 0.038 c	27.82 ± 0.34 c	0.63 ± 0.013 d	0.02 ± 0.001 e
*P. patula* (413)	91.90 ± 11.90 b	5.339 ± 0.215 d	5.719 ± 0.024 b	0.94 ± 0.05 f	1.41 ± 0.017 b	0.24 ± 0.01 c
*P. hederifolia var. hederifolia* (407)	54.15 ± 1.64 c	16.48 ± 0.153 b	Traces	7.68 ± 0.08 e	1.03 ± 0.002 c	0.06 ± 0.000 d
*P. angulata* (401)	33.18 ± 1.43 c	8.829 ± 0.032 c	1.389 ± 0.009 d	31.94 ± 3.03 b	2.10 ± 0.16 a	0.09 ± 0.01 d
Quercetin				5.29 ± 0.39 e	0.08 ± 0.002 e	0.09 ± 0.003 d
Epicatechin				14.33 ± 0.29 d	0.11 ± 0.005 e	0.07 ± 0.004 d

The values represent the mean and standard deviation for three independent samples. Different letters in the same column for each type of tissue mean significant differences (p < 0.05)

Table 2 shows the flavonoid contents found in the different tissues of the analyzed species of *Physalis*. Significant tissue and species-dependent variations were found. The foliar flavonoid contents explained between 7.55 and 56.86 % of total phenolics, the highest level was found for *P. angulata* (23.036 mg/g dt). Flavonoid contents in fruits represented between 0.0 and 16.91 % of total phenolics, the fruits of *P. subulata* showed the highest level (5.462 mg/g dt). Flavonoid levels in calyces (representing between 3.08 and 30.43 % of total phenolics) ranged from 3.67 mg/g dt in *P. solanacea* (sample 412) to 39.63 mg/g dt in *P. subulata*.

Species- and tissue-dependent variations were also found in the phenolic acid contents (Table 2). In leaves, phenolic acids corresponded to between 2.11 and 16.55 % of total phenolics and *P. angulata* leaves showed the highest level (6.7 mg/g dry tissue). In fruits, phenolic acids represented between 0.0 and 21.25 % of total phenolics; the fruits of *Physalis angulata* were the richest in these type of phenolics (7.84 mg/g dt). Excepting the calyces of *P. hederifolia* var. *hederifolia*, which practically did not accumulate phenolic acids, these compounds explained from between 2.86 and 28.63 % of total phenolics. The content of phenolic acids in the calyces of *P. subulata* (50.57 mg/g dt) was outstanding.

### Phenolic profiles

Compounds **22** (λ_max_: 255, 264sh, 294sh, 355), **F7** (λ_max_: 255, 264sh, 294sh, 355), and **C11** (λ_max_: 255, 264sh, 294sh, 355) were proposed as rutin because of its spectral data corresponded to those reported by Campos and Markham (2007) for that flavonol (λ_max_: 255, 264sh, 294sh, 355).

**Fig. 1:**
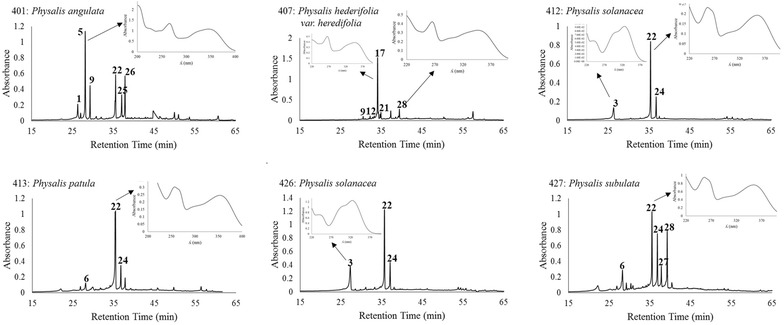
HPLC chromatograms and UV spectra of the major foliar phenolics of five species of *Physalis*. The HPLC chromatograms were registered at 260 nm and the UV spectra of the resolved compounds were obtained from 200 to 400 nm. The number of compounds corresponds to those of Table 3

**Fig. 2:**
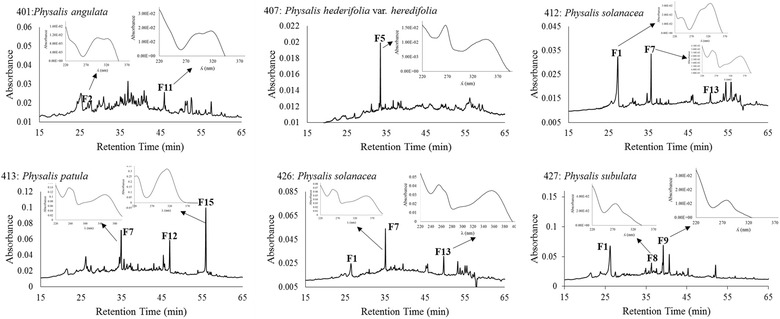
HPLC chromatograms and UV spectra of major fruit phenolics of five species of *Physalis*. The HPLC chromatograms were registered at 260 nm and the UV spectra of the resolved compounds were obtained from 200 to 400 nm. The number of compounds corresponds to those of Table 3

**Fig. 3:**
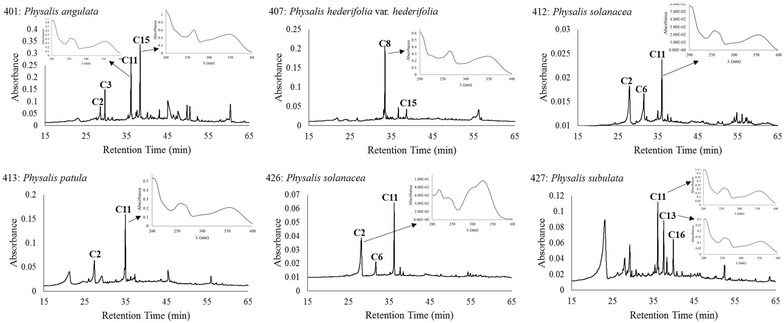
HPLC chromatograms and UV spectra of major calyx phenolics of five species of *Physalis*. The HPLC chromatograms were registered at 260 nm and the UV spectra of the resolved compounds were obtained from 200 to 400 nm. The number of compounds corresponds to those of Table 3

The HPLC–DAD analysis revealed a total of 28 phenolic compounds in the leaves of the five species of *Physalis* analyzed (the respective chromatograms are shown in Fig. 1). Twenty of those compounds were flavonols (among them, 14 were kaempferol-3-*O*-glycosides and 5 were quercetin-3-*O*-glycosides) and 8 were phenolic acids. The respective retention times and λ_max_ are displayed in Table 3. The HPLC chromatograms of fruits (Fig. 2) revealed a total of 16 compounds: 11 phenolic acids, 3 flavonols (2 quercetin-3-*O*-glycosides and 1 kaempferol-3-*O*-glycoside), and 2 dihydroflavonols (retention times and λ_max_ are displayed in Table 3). A total of 16 phenolic compounds were unveiled by the chromatograms of calyces (Fig. 3), among which 12 were flavonols (10 kaempferol-3-*O*-glycosides and 2 quercetin-3-*O*-glycosides) and 4 were phenolic acids (Table 3). The concentration of each phenolic compound found in the different analyzed species is shown in Table 3.

**Table 3:** Phenolic compounds found in the leaves, fruits, and calyces of five wild species of *Physalis*

Number of compound	RT (min)	λ_max_	Type of phenolic compound	Species
Leaves				
**1**	26.50 ± 0.11	256, 267sh, 319, 360sh	Flavonol	*P. angulata* (2.476)
**2**	22.57 ± 0.36	240sh, 296sh, 326	Phenolic acid	*P. subulata* (2.184)
**3**	27.37 ± 0.07	240sh, 296sh, 325	Phenolic acid	*P. solanacea* (412: 2.288; 426: 4.671)
**4**	27.23 ± 0.11	256, 266sh, 347	Quercetin-3-*O*-glycoside	*P. angulata* (1.544)
**5**	28.41 ± 0.02	243sh, 265, 317sh, 345	Kaempferol-3-*O*-glycoside	*P. angulata* (5.094)
**6**	28.36 ± 0.32	233sh, 291sh, 324	Phenolic acid	*P. angulata* (1.281) *P. patula* (2.467) *P. hederifolia* var *hederifolia* (1.242) *P. subulata* (3.381)
**7**	28.73 ± 0.05	227sh, 243sh, 265, 344	Kaempferol-3-*O*-glycoside	*P. subulata* (1.522)
**8**	29.63 ± 0.24	243sh, 290sh, 322	Phenolic acid	*P. patula* (1.586)
**9**	29.65 ± 0.09	227sh, 265, 343	Kaempferol-3-*O*-glycoside	*P.angulata* (2.329) *P. hederifolia* var. *hederifolia* (1.412) *P. subulata* (1.524)
**10**	31.17 ± 0.07	229sh, 268sh, 308	Phenolic acid	*P. angulata* (1.417)
**11**	31.87 ± 0.00	231sh, 267sh, 312	Phenolic acid	*P. angulata* (1.268)
**12**	32.21 ± 0.05	256, 266sh, 353	Quercetin-3-*O*-glycoside	*P. hederifolia* var. *hederifolia* (1.403)
**13**	32.43 ± 0.03	226sh, 295sh, 312	Phenolic acid (probably *p*-coumaric acid)	*P. angulata* (1.341)
**14**	32.93 ± 0.00	265, 318sh, 354	Kaempferol-3-*O*-glycoside	*P. hederifolia* var. *hederifolia* (1.331)
**15**	33.29 ± 0.00	228sh, 266, 320sh, 343	Kaempferol-3-*O*-glycoside	*P. hederifolia* var. *hederifolia* (1.515)
**16**	33.82 ± 0.00	228sh, 265, 317sh, 345	Kaempferol-3-*O*-glycoside	*P. hederifolia* var. *hederifolia* (1.897)
**17**	34.16 ± 0.00	227sh, 244sh, 265, 318sh, 345	Kaempferol-3-*O*-glycoside	*P. hederifolia* var. *hederifolia* (6.868)
**18**	34.29 ± 0.00	228sh, 265, 317sh, 346	Kaempferol-3-*O*-glycoside	*P. hederifolia* var. *hederifolia* (1.538)
**19**	34.58 ± 0.00	236sh, 270sh, 290sh, 324	Phenolic acid	*P. angulata* (1.397)
**20**	34.75 ± 0.10	265, 294sh, 344	Kaempferol-3-*O*-glycoside	*P. hederifolia* var. *hederifolia* (1.583)
**21**	34.90 ± 0.03	228sh, 245sh, 266, 345	Kaempferol-3-*O*-glycoside	*P. hederifolia* var. *hederifolia* (1.631)
**22**	35.69 ± 0.20	255, 264sh, 294sh, 355	Quercetin-3-*O*-glycoside (rutine)	*P. angulata* (5.175) *P. patula* (5.431) *P. solanacea* (412: 4.388; 426: 6.882) *P. subulata* (5.892)
**23**	34.85 ± 0.60	255,266sh, 296sh, 355	Quercetin-3-*O*-glycoside	*P. patula* (3.627)
**24**	36.24 ± 0.34	256, 266sh, 296sh, 353	Quercetin-3-*O*-glycoside	*P. patula* (2.421) *P. solanaceous* (412: 2.154, 426: 4.682) *P. subulata* (4.599)
**25**	36.47 ± 0.16	227sh, 265, 294sh, 346	Kaempferol-3-*O*-glycoside	*P. angulata* (2.837)
**26**	37.40 ± 0.22	227sh, 265, 294sh, 346	Kaempferol-3-*O*-glycoside	*P. angulata* (3.577) *P. patula* (1.879)
**27**	38.20 ± 0.08	265, 293sh, 343	Kaempferol-3-*O*-glycoside	*P. subulata* (2.597)
**28**	39.44 ± 0.12	264, 293sh, 346	Kaempferol-3-O-glycoside	*P. hederifolia var. hederifolia* (2.032) *P. subulata* (3.977)
Fruits				
**F1**	26.648 ± 0.489	293sh, 325	Phenolic acid	*Physalis solanacea* (412: 2.773; 426: 1.253) *P. subulata* (2.470)
**F2**	27.389 ± 0.187	296sh, 322	Phenolic acid	*P. angulata* (1.534) *P. patula* (1.144) *P. subulata* (1.787)
**F3**	29.821 ± 0.000	292sh, 326	Phenolic acid	*P. angulata* (1.536)
**F4**	30.716 ± 0.000	283sh, 311	Phenolic acid	*P. patula* (1.163)
**F5**	33.488 ± 0.000	226sh, 265, 346	Kaempferol-3-*O*-glycoside	*P. hederefolia var hederifolia* (4.807)
**F6**	34.47 ± 0.000	291sh, 311	Phenolic acid	*P. patula* (1.232)
**F7**	35.138 ± 0.323	255, 264sh, 294sh, 355	Quercetin-3-*O*-glycoside	*P. patula* (1.392) *P. solanacea* (412: 2.320, 426: 1.282) *P. subulata* (1.765)
**F8**	37.55 ± 0.000	281, 324sh	Dihydroflavonol	*P. subulata* (1.765)
**F9**	39.193 ± 0.000	277, 303sh	Dihydroflavonol	*P. subulata* (1.932)
**F10**	45.69 ± 0.000	286sh, 312	Phenolic acid	*P. patula* (1.134)
**F11**	45.954 ± 0.000	283sh, 317	Phenolic acid	*P. angulata* (1.593)
**F12**	46.937 ± 0.000	291sh, 311	Phenolic acid	*P. patula* (1.282)
**F13**	50.091 ± 0.397	255, 267sh, 296sh, 355	Quercetin-3-*O*-glycoside	*P. solanaceous* (412: 2.322; 426: 1.217)
**F14**	52.628 ± 0.000	292sh, 321	Phenolic acid	*P. angulata* (1.610)
**F15**	55.928 ± 0.000	290sh, 312	Phenolic acid	*P. patula* (1.429)
**F16**	57.497 ± 0.000	281sh, 318	Phenolic acid	*P. angulata* (1.573)
Calyces				
**C1**	23.055 ± 0.054	246sh, 290sh, 322	Phenolic acid	*Physalis angulata* (1.389) *Physalis subulata* (25.085)
**C2**	27.855 ± 0.287	246sh, 292, 326	Phenolic acid	*Physalis solanacea* (412: 2.034; 426: 3.100) *P. patula* (3.085) *Physalis subulata* (12.132)
**C3**	28.574 ± 0.000	226sh, 266, 287sh, 347	Kaempferol-3-*O*-glycoside	*Physalis angulata* (1.549)
**C4**	29.180 ± 0.040	242sh, 286sh, 322	Phenolic acid	*Physalis patula* (2.634) *Physalis subulata* (13.352)
**C5**	29.641 ± 0.000	228sh, 265, 345	Kaempferol-3-*O*-glycoside	*Physalis angulata* (1.558)
**C6**	31.400 ± 0.317	246sh, 316	Phenolic acid	*Physalis solanacea* (412: 2.020; 426: 2.704)
**C7**	33.172 ± 0.036	229sh, 266, 318sh, 344	Kaempferol-3-*O*-glycoside	*Physalis hederifolia var. hederifolia* (3.084)
**C8**	33.572 ± 0.146	227sh, 2656, 316sh, 346	Kaempferol-3-*O*-glycoside	*Physalis hederifolia* var. *hederifolia* (4.214)
**C9**	33.605 ± 0.000	229sh, 266, 319sh, 344	Kaempferol-3-*O*-glycoside	*Physalis hederifolia var. hederifolia* (3.023)
**C10**	34.016 ± 0.000	225sh, 265, 340	Kaempferol-3-O-glycoside	*Physalis hederifolia var. hederifolia* (3.017)
**C11**	35.990 ± 0.064	255, 264sh, 294sh, 355	Quercetin-3-*O*-glycoside, (Rutin)	*Physalis angulata* (2.087) *Physalis solanacea* (412: 2.032; 426: 2.671) *Physalis patula* (3.080) *Physalis subulata* (13.885)
**C12**	37.407 ± 0.135	267sh, 265, 293sh, 340	Kaempferol-3-*O*-glycoside	*Physalis angulata* (1.320) *Physalis patula* (2.124)
**C13**	37.55 ± 0.000	256, 266sh, 295, 352	Quercetin-3-*O*-glycoside	*Physalis subulata* (13.278)
**C14**	38.606 ± 0.000	265, 293sh, 346	Kaempferol-3-*O*-glycoside	*Physalis solanaceous* (412: 1.646; 426: 2.631)
**C15**	38.437 ± 0.175	225sh, 265, 291sh, 346	Kaempferol-3-*O*-glycoside	*Physalis angulata* (2.335) *Physalis hederifolia var. hederifolia* (3.146)
**C16**	39.897 ± 0.000	226sh, 265, 345	Kaempferol-3-*O*-glycoside	*Physalis subulata* (12.469)

Figures in brackets mean concentration (mg/g dry tissue)

### Cluster analysis

**Fig. 4:**
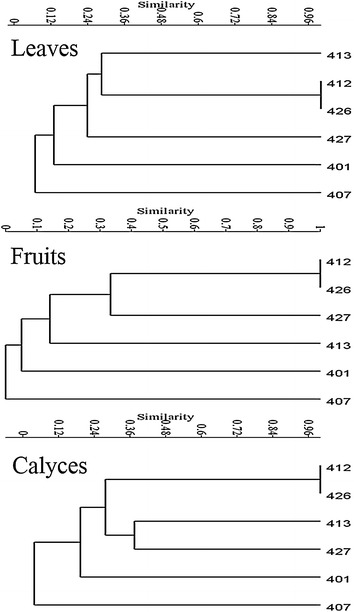
Results of the cluster analysis based on the phenolic profiles of five species of *Physalis.* The dendrogram was generated with the Paired Group Algorithm and the Jaccard’s Similarity Measure, for comparing foliar, fruit and calyx phenolic profiles for five wild species of *Physalis*. The reference number of samples corresponds to those of Table 1

The results of the cluster analysis based on the matrices constructed with each foliar, fruit, and calyx phenolic profile of each sample of *Physalis* are displayed in Fig. 4. All species were discriminated one from each other at levels of similarity from around 0.06 (Jaccard similarity measure, according to the foliar phenolic profiles) to around 0.42 (according to the calyx phenolic profiles).

### Antioxidant assays

The results of the free radical scavenging activity, total antioxidant capacity, and iron reducing power are shown in Table 2. Specific and tissue dependent variations were found in the three antioxidant assays.

### Correlation analysis

While the kinetic evaluation of all the antioxidant assays was highly related to flavonoid contents (0.9731 < r < 0.999), the correlation analysis revealed lower associations between the antioxidant properties and the phenolic, flavonoid, and phenolic acid contents in the samples (0.224 between calyx total flavonoids and calyx IC_50_ <Pearson correlation value <0.856 between calyx total phenolics and calyx IC_50_).

### Principal component analysis

**Fig. 5:**
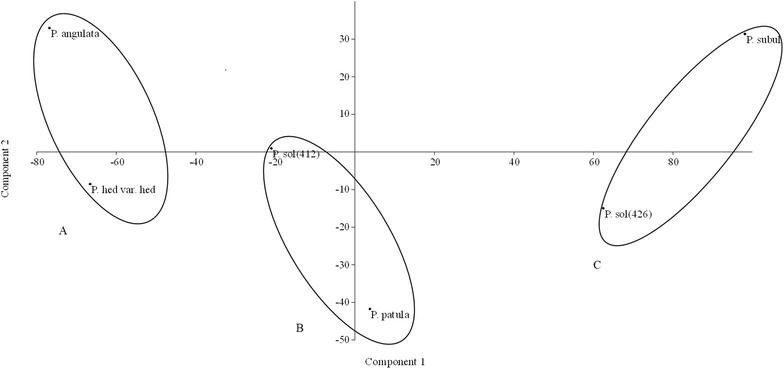
Results of a principal component analysis (PCA). The contents of total phenolics, flavonoids and phenolic acids, along with the estimations of antioxidant capacities of five wild species of *Physalis* were analyzed using PCA.* Group A*: samples collected in August;* group B*: samples collected in September;* C*: samples collected in October

The results of a PCA, based on the quantitative determinations (phenolic, flavonoid, and phenolic acid contents and the estimation of antioxidant capacity by three methods) are showed in the Fig. 5. Three principal components accounted for 93.77 % of total variance, being the calyx phenolic content (PC1) the main one, taking 73.467 %, with the highest relative discriminating power (eigenvalue 4860.76). The calyx antiradical activity (PC2) explained the 12.425 % of total variance (eigenvalue 822.079), and the foliar phenolic content, the 7.88 % (eigenvalue 521.505).

## Discussion

### Phenolic, flavonoid, and phenolic acid contents

Phenolic compounds contribute to the organoleptic and antioxidant properties of foods and they are among the most important nutraceuticals of interest in food industry (Tapas et al. 2008). Some, like phenolic acids, play important roles in fruit maturation, prevention of enzymatic browning, and food preservation (Robbins 2003). Phenolics also represent important tools for food tracing and to prove authenticity of foods (Zimmermann and Galensa 2007). The understanding of their abundance, diversity, and distribution in the plant kingdom and in the different plant tissues offers the opportunity of developing new drugs to improve human health, and to develop new varieties with enhanced accumulation of these compounds through breeding programs.

The potential of different species to synthesize and accumulate flavonoids, and phenolics in general, is the result of the interaction of genetic and environmental factors (Veit et al. 1995) and different species of plants can synthesize and accumulate widely variable levels (Diaz et al. 2012). Aside from the differences in the flavonoid levels due to diverse environmental conditions of growing and to the specific variations, differences between tissues can be expected as the result of the differential regulation of the genic expression in well differentiated cells.

The total phenolic concentrations observed in the leaves and fruits of the analyzed species of *Physalis* were comparable to those reported by Wang and Lin (2000) in the leaves (30.9–129.2 mg/g dry matter) and fruits (9.16–23.10 mg/g dry matter basis) of several cultivars of black raspberry, red raspberry, and strawberries, all considered as important source of phenolics. To our knowledge, this is the first report of phenolic contents in calyx for the species here analyzed of *Physalis* and reveals that these structures can accumulate relevant phenolic levels. The present results indicate that the species analyzed of *Physalis* are important sources of total phenolics, mainly accumulated in leaves and calyces.

Comparatively, the found foliar flavonoid contents were lower than those reported by Wu et al. (2009) for the leaves of *P. peruviana* (37.39–226.19 mg/g dry extract, depending on the type of solvent used for the extraction). Excepting *P. angulata*, in whose fruits no flavonoids were found, the fruit flavonoid contents found in the present study (Table 2) were higher than that reported for ripe fruits of *Lycium barbarum* (Fructus Lycii) (0.72 mg/g dry fruit), whose importance as source of antioxidants has been reported by Le et al. (2007). The flavonoid levels found in the calyces of *P. subulata* (Table 2) can be comparable to that reported for the aqueous extract of *P. peruviana* leaves (37.39 mg/g) by Wu et al. (2009). Flavonoids were mainly accumulated in leaves and calyces.

Studies on determinations of phenolic acids in species of *Physalis* have been mainly focused on fruits (Rockenbach et al. 2008); however, the current results reveal that leaves and calyces of the species of *Physalis* can accumulate these phenolics at similar or even higher levels than fruits (Table 2).

### Phenolic profiles

Quercetin derivatives, kaempferol derivatives, and phenolic acids were the major phenolic compounds synthesized by the five species analyzed of *Physalis*, and were accumulated with a species-specific tendency.

The leaves of *P. angulata* accumulated the highest number of phenolics (12 compounds) and those of *P. solanacea* the lowest (3 compounds) (Table 3). Rutin (compound **22**) was found in all species (as one of the dominant compounds), except in *P. hederifolia* var. *hederifolia*, whose leaves were rich in kaempferol-3-*O*-glycosides (9 among 11 compounds were kaempferol derivatives). Rutin has also been reported in aerial parts of *Physalis orizabae* (Maldonado et al. (2012). Myricetin-3-*O*-neohesperidoside (λ_max_: 266, 299sh, 356) was reported in methanol extracts of leaves of *P. angulata* by Ismail and Alam (2001); however, the current results revealed no myricetin derivatives for any of the analyzed species.

In fruits, phenolic acids were the dominant compounds (Table 3), except for *P. hederifolia* var. *hederifolia*. A similar dominance has been reported for the fruits of *P. peruviana* (Rockenbach et al. 2008). Relevant interspecific differences were observed in the phenolic composition, for example, for the fruits of *P. hederifolia* var. *hederifolia* the only phenolic compound found was one kaempferol-3-*O*-glycoside (**F5**) (Fig. 2), this contrasts with the composition found for the other four species and with the results reported for *P. peruviana* (Rockenbach et al. 2008). Rutin and myricetin have been found in the fruits of *P. peruviana*, rutin being present as a predominant compound (Licodiedoff et al. 2013). Rutin (compound **F7**) was also present in the fruits of *P. patula*, *P. solanacea*, and *P. subulata*, and in the two first species it was one of the dominant compounds (Fig. 2). However, none of the spectral data found in the present study corresponded to those of myricetin reported by Campos and Markham (2007) (λ_max_: 252, 263sh, 298sh, 371) or by Mabry et al. (1970) (λ_max_: 254, 272sh, 301sh, 374). The fruits of *P. subulata* contained dihydroflavonoids (**F8** and **F9**), which are less common phenolics in plant kingdom than flavonoids. The concentrations of **F8** and **F9** were similar to those of most phenolic acids (Table 3). Dihydroflavonoids can be important inhibitors of tumor development, antioxidant, and analgesic (Wong and Rabie 2009). The fruits of *P. patula* accumulated the highest number of phenolic compounds (seven).

In calyces of *P. hederifolia* var. *hederifolia*, as in its leaves and fruits, the dominant compounds were kaempferol-3-*O*-glycosides, and this was the only species in whose calyces, rutin (compound **C11**) was missing. Rutin has also been reported in the calyx of *P. solanacea* by Pérez-Castorena et al. (2013). The number of compounds accumulated in the calyces varied from 4 in *P. solanacea* and *P. patula* to 7 in *P. subulata*.

The concentrations of the individual phenolics were variable between species and between tissues. The highest level was found for compound **C1,** a phenolic acid accumulated in the calyces of *P. subulata* (Table 3). Comparatively, the individual phenolic acid concentrations of fruits and calyces were higher than the concentrations reported by Rockenbach et al. (2008) for the fruits of *Physalis peruviana*.

### Cluster analysis

The results of the cluster analysis suggests that the phenolic profiles obtained by HPLC–DAD (Fig. 4) were species-specific, independent from location and time of collection, as indicated for the two samples of *P. solanacea* (Jaccard similarity value of 1), in such a way that each species can be typified by its phenolic profile. These phenol profiles can be important specific chemotaxonomic markers for *Physalis* and may contribute to solve taxonomic controversies around the delimitation of species mentioned by several authors (Whitson and Manos 2005). The species-specific feature of the profiles can be used also as a tool to typify, trace, and define the authenticity of fruits and preparations of these species. The chemical diversity revealed for the five wild analyzed species of *Physalis* can be a starting point to improve the quality of fruit of the cultivated species of the genus, and could represent relevant allelic forms. Our results corroborate previous reports about the specificity of phenolic profiles (Almaraz-Abarca et al. 2013).

### Antioxidant properties and correlation analysis

All samples displayed important antioxidant properties. As scavengers of the free radical DPPH highlighted the fruits of *P. hederifolia* var. *hederifolia* (Table 2). All extracts showed higher scavenging activity than that reported by Chang et al. (2008) for the fruits of *P. peruviana* (EC_50_ value around 300 µg/mL). All extracts showed higher power to reduce Mo (VI) to Mo (V) than quercetin and epicatechin (Table 2), both considered as powerful antioxidants (Tapas et al. 2008). Leaves of *P. subulata* displayed until 44-fold higher values of TAC than any of two standards. All the foliar extracts showed higher iron reduction potential than the standards quercetin and epicatechin (Table 2). However, the highest value, which was reached by the extract of calyces of *P. solanacea* (426) was lower than the value reported by Diaz et al. (2012) for the fruits of *P. alkekengi* (IC_50_ = 36.58 mg/g dry weight).

The variations found among the two samples of *P. solanacea* suggest that as the phenolic profiles were the same for both samples (Figs. 1, 2, 3), plant age caused variations in the concentrations of phenolics (Tables 2, 3), all which affected the antioxidant properties of tissues. These kind of effects have been reported for other plant species (Del Baño et al. 2003). In fruits, the accumulation of other antioxidants, like ascorbic acid, could affect the antioxidant properties. Synthesis of ascorbic acid has been reported in the fruits of *P. peruviana* (Rop et al. 2012) and may occur also in the species of *Physalis* here analyzed.

The low association between the antioxidant properties and the phenolic, flavonoid, and phenolic acid contents found in the present study agree with the reports of other authors (Morais et al. 2011; Barriada-Bernal et al. 2014), who suggested that the antioxidant potential of a given total extract depends not only on the amount of phenolic compounds present in it but also on the types of phenolics accumulated, their proportions, as well as the presence of compounds of different chemical nature.

### Principal component analysis

The results of the PCA revealed a tendency of grouping based on the developmental stage (Fig. 5), group A included samples collected in August (*P. hederifolia* var. *hederifolia* and *P. angulata*), group B was formed by samples collected in September (*P. patula* and *P. solanacea* 412), and group C was formed by samples collected in October (*P. solanacea* 426 and *P. subulata*). These results reveal the effect of time of collection and the associated developmental stage, on the phenolic, flavonoid, and phenolic acids contents and the antioxidant properties of different tissues of five wild species of *Physalis*. Systematic studies concerning the seasonal variation of phenolics of the species of *Physalis* are needed, as it has been done for some species of *Betula* (Raal et al. 2015), and concerning the developmental variation, as it has been carried out for *Rosmarinus officinalis* (Del Baño et al. 2003). Those studies would contribute to identify the best collecting time and developmental stage to obtain the highest concentration of some phenolic compounds and the environmental conditions in which they are produced.

## Conclusions

The wild analyzed species of *Physalis* synthesize important amounts of diverse phenolic compounds. The phenolic profiles of leaves, fruits, and calyces were species-specific; this reveals phenolic patterns as significant specific chemomarkers in the genus. The dominant phenolic compounds accumulated in leaves and calyces were 3-*O*-glycoside derivatives of kaempferol, and the dominant ones in fruits were phenolic acids; all these compounds are recognized for their important activities as antioxidants. Because of their phenolic compositions, *P. angulata, P. patula, P. hederifolia var. hederifolia, P. solanacea, and P. subulata* may be considered as relevant potential sources of natural antioxidants to be used in the food and pharmaceutical industries. The present phenolic fingerprinting contributes to generate a better knowledge for using all the potential of these wild species of plants. The chemical variability of wild species of *Physalis* offers the opportunity for domesticating and developing new cultivars with enhanced accumulation of phenolics having positive effects on human health. The results inform about the importance of consuming these plant species and support the use of calyces and leaves in traditional medicine. The not always high correlation between phenolic or flavonoid contents and antioxidant activities suggests the relevance of the phenolic profiles, besides the contents of phenolics, in determining these activities.

## Abbreviations

AAE: ascorbic acid equivalents
CE: *p*-coumaric acid equivalents
DAD: diode array detector
DPPH: 2,2-diphenyl-1-picrylhydrazyl
dt: dry tissue
EC_50_: efficient concentration at 50 %
GAE: gallic acid equivalents
HPLC: high performance liquid chromatography
IC_50_: half maximal inhibitory concentration
NE: naringenin equivalents
PCA: principal component analysis
PC1: principal component 1
PC2: principal component 2
QiE: quercitrin equivalents
TAC: total antioxidant capacity
UV: ultraviolet
